# The long noncoding RNA Neat1 is required for mammary gland development and lactation

**DOI:** 10.1261/rna.047332.114

**Published:** 2014-12

**Authors:** Laura Standaert, Carmen Adriaens, Enrico Radaelli, Alexandra Van Keymeulen, Cedric Blanpain, Tetsuro Hirose, Shinichi Nakagawa, Jean-Christophe Marine

**Affiliations:** 1 Center for the Biology of Disease, Laboratory for Molecular Cancer Biology, VIB, Leuven 3000, Belgium; 2 Center for Human Genetics, Laboratory for Molecular Cancer Biology, KULeuven, Leuven 3000, Belgium; 3 Center for the Biology of Disease, Histopathology Lab, VIB, Leuven 3000, Belgium; 4 Center for Human Genetics, Histopathology Lab, KULeuven, Leuven 3000, Belgium; 5 Université Libre de Bruxelles, IRIBHM, Brussels 1070, Belgium; 6 Institute for Genetic Medicine, Hokkaido University, Sapporo 060-0815, Japan; 7 RNA Biology Laboratory, RIKEN, Saitama 351-0198, Japan

**Keywords:** NEAT1, long noncoding RNA, paraspeckles, mammary gland development

## Abstract

The lncRNA Neat1 is an essential architectural component of paraspeckle nuclear bodies. Although cell-based studies identified Neat1-paraspeckles as key regulators of gene expression through retention of hyperdited mRNAs and/or transcription factors, it is unclear under which specific physiological conditions paraspeckles are formed in vivo and whether they have any biological relevance. Herein, we show that paraspeckles are assembled in luminal epithelial cells during mammary gland development. Importantly, genetic ablation of *Neat1* results in aberrant mammary gland morphogenesis and lactation defects. We provide evidence that the lactation defect is caused by a decreased ability of Neat1-mutant cells to sustain high rates of proliferation during lobular-alveolar development. This study is the first to assign an important biological function to the lncRNA Neat1 and to link it to the presence of paraspeckles nuclear bodies in vivo.

## INTRODUCTION

Although long noncoding (lnc) RNAs are emerging as key regulators of various cellular processes based on in vitro transfection studies, direct genetic evidence of their functional significance in vivo remains elusive. Gain and loss of function studies in cell-based in vitro systems have indicated that lncRNAs may play essential roles in as diverse and essential cellular processes as the regulation of chromatin states, maintenance of cellular identity, cell cycle, or translation (Rinn and Chang 2012; Ulitsky and Bartel 2013; Fatica and Bozzoni 2014). These initial exciting studies, however, warrant confirmation of the biological importance of the lncRNAs in more physiological and pathophysiological settings (Li and Chang 2014).

Surprisingly, of the few lncRNA mutant mice generated to date, most did not exhibit any obvious phenotypes. For instance, mice lacking the abundant lncRNA Malat1, which localizes to nuclear bodies known as speckles and is involved in key cellular functions such as mRNA splicing, are viable and fertile (Eissmann et al. 2012; Nakagawa et al. 2012; Zhang et al. 2012). Similarly, mice lacking Neat1 develop normally and are indistinguishable from their wild-type (WT) littermates with respect to growth, viability, and apparent behavior (Nakagawa et al. 2011). Neat1 is another very abundant lncRNA required for the formation of another subclass of nuclear bodies, paraspeckles (Chen and Carmichael 2009; Clemson et al. 2009; Sunwoo et al. 2009). Several studies have dissected in detail the dynamics of paraspeckle assembly and their cellular functions in cultured cells (Chen and Carmichael 2009; Clemson et al. 2009; Mao et al. 2011; Passon et al. 2012; Hirose et al. 2014; Imamura et al. 2014). These biochemical and cellular studies have implicated paraspeckles in the regulation of gene expression through the sequestration of specific transcription factor(s) (Hirose et al. 2014; Imamura et al. 2014) and of A-I hyperedited mRNAs (Prasanth et al. 2005; Fox and Lamond 2010; Nakagawa and Hirose 2012). It has been proposed that through their ability to integrate transcription and post-transcriptional events, paraspeckles are likely to control several key cellular processes such as cellular differentiation (Chen and Carmichael 2009; Nakagawa and Hirose 2012). The absence of obvious phenotypic abnormalities in mice lacking Neat1 has, however, challenged this view and questioned the biological significance of Neat1 and paraspeckles.

The mouse *Neat1* gene produces two isoforms, 3.2 kb Neat1_1 and 20 kb Neat1_2 (Hutchinson et al. 2007). Whereas paraspeckle formation requires expression of the Neat1_2 isoform (Naganuma et al. 2012), it is not sufficient for paraspeckle assembly in vivo. Although high levels of Neat1_2 are detected in most mouse and human tissues by RT-qPCR, paraspeckles have so far only been observed in vivo in chief cells of the gastric glands (Nakagawa et al. 2011). Consistent with Neat1 being essential for their assembly, paraspeckles are no longer observed in the stomach of Neat1 knockout (KO) mice (Nakagawa et al. 2011). However, histological and functional analyses did not reveal any phenotypic consequences associated with the absence of Neat1 and paraspeckles in these cells (Nakagawa et al. 2011).

Together these observations indicate that paraspeckles either do not play any major relevant biological functions under normal physiological conditions or that paraspeckles are assembled and play critical biological functions in response to specific stress signals/challenges and/or in very few—yet to be identified—specialized cell types/tissues.

In this study, we show that Neat1-containing paraspeckles are formed in luminal cells during mammary gland development and that, importantly, Neat1 is required for mammary gland branching morphogenesis, lobular-alveolar development, and lactation. This study therefore unequivocally identifies the first physiological function of one of the most abundant long noncoding RNAs, Neat1, in a key biological process, namely mammary gland development and lactation.

## RESULTS AND DISCUSSION

### Lobuloalveogenesis and lactation are compromised in absence of Neat1

**FIGURE 1.**
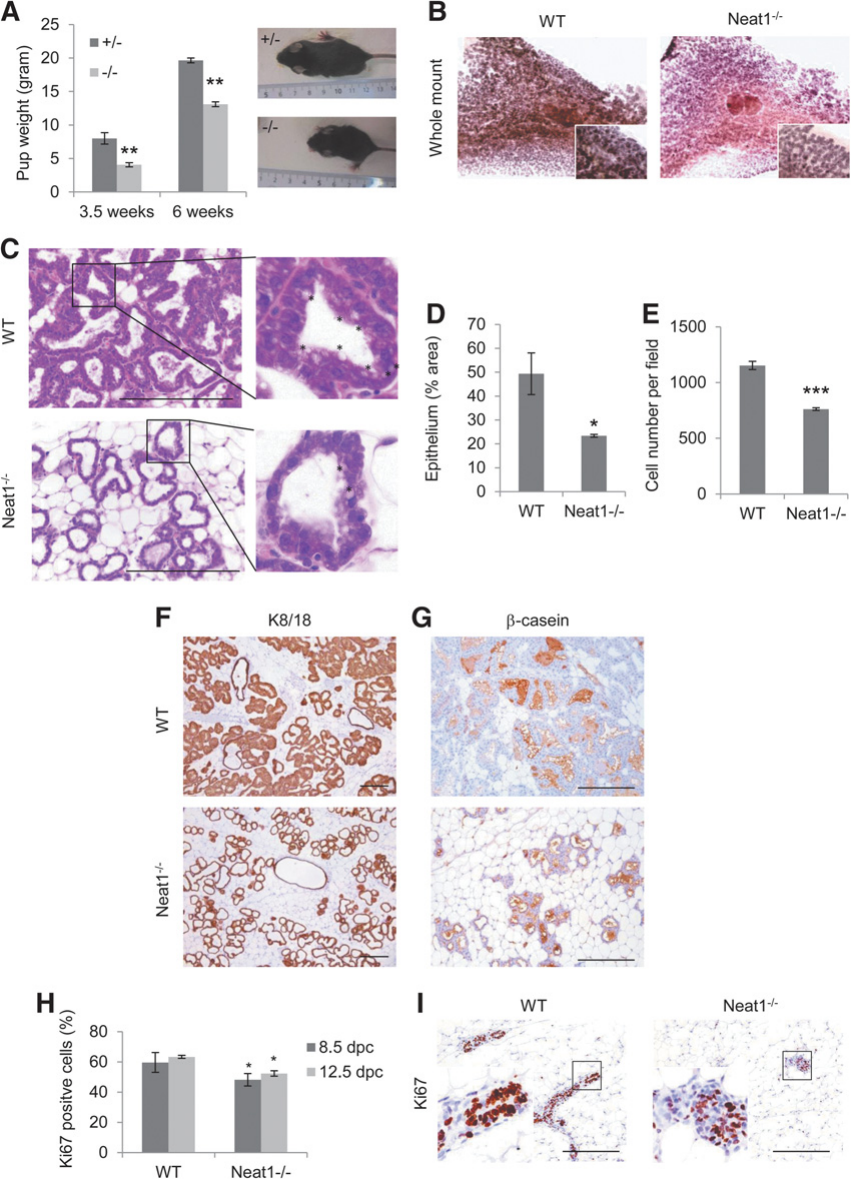
Loss of Neat1 leads to lobuloalveolar development and lactation defects. (*A*) Average weight of offspring from Neat1 heterozygous (+/− mother) and Neat1 KO (−/− mother) female before (3.5 wk) and after weaning (6 wk) and representative images. Statistical significance determined by two-sided *t*-test. (**) *P* = 0.0044 at 3.5 wk and (**) *P* = 0.0002 at 6 wk. (*B*) Whole-mount hematoxylin staining of wild-type (WT) and Neat1 KO (Neat1^−/−^) lactating inguinal mammary glands at 2 d post-parturition. (*C*) H&E staining on sections obtained from the inguinal mammary glands contralateral to the ones described in *B*. Comparative quantification of (*D*) luminal alveolar compartment (K8/18 immunoreactivity); (*) *P* = 0.028 and (*E*) cell number (DAPI stain) in the lactating mammary gland of wild-type versus Neat1 KO females; (***) *P* = 0.0003. Statistical significance determined by two-sided *t*-test. (*F*) Immunohistochemical staining of cytokeratin 8/18 on consecutive sections of samples in *C*. (*G*) Immunohistochemical staining of β-casein on consecutive sections of samples in *C*. (*H*) Comparative quantification of Ki67-positive cells relative to total cell number in mammary glands of pregnant females at 8.5 (*) *P* = 0.04 and 12.5 (*) *P* = 0.01 d post-coitum. (*I*) Immunohistochemical staining of Ki67 on sections obtained from inguinal mammary gland 8.5 d post-coitum of wild-type and Neat1^−/−^ females.

We noticed that only a minority of pups (24%) derived from homozygous Neat1 KO females survive beyond 5 d after birth; all these offspring contain a smaller amount of milk in their stomach and are reduced in size and weight compared with pups fed by wild-type or Neat1 heterozygous (Neat1^+/−^) mothers (Fig. 1A). In contrast, >85% of the pups from the WT and Neat1 heterozygous (Neat1^+/−^) mothers are fed, gain weight normally, and survive until weaning age. Importantly, pups from Neat1 KO females also gain weight normally and survive until weaning age when fed by foster mothers. These observations indicate that Neat1-mutant mothers are compromised in their ability to nurture their pups. This phenotype could be a consequence of qualitative and/or quantitative defect(s) in milk production.

To characterize this phenotype in more detail, we examined lactating Neat1 KO mammary glands microscopically. In contrast to Neat1^+/+^ mammary glands, Neat1^−/−^ glands exhibit markedly fewer alveoli and a greatly reduced epithelial content at day 2 of lactation as shown in whole-mount preparations (Fig. 1B) and hematoxylin/eosin (H&E)-stained tissue sections (Fig. 1C). The decrease in epithelium content was quantified (Fig. 1D) and shown to be a consequence of a decrease in epithelial cell number, rather than cell size (Fig. 1E), in particular of the cytokeratin 8/18 (K8/18)-positive luminal cell compartment (Fig. 1F). Importantly, lactating Neat1^−/−^ mammary glands are positive for the lactation markers β-casein and whey acidic protein (Fig. 1G and data not shown) and only exhibited a partial loss of cytoplasmic droplets indicative of active milk secretion (Fig. 1C). Neat1 is therefore not required for milk production per se or for its secretion by the luminal alveolar epithelial cells. Together these data indicate that the reduced nursing ability of Neat1 KO females is due to a decrease in the number of K8/18-positive/milk-producing cells rather than a block in functional differentiation of epithelial cells into milk-producing cells.

To gain insight into the cellular basis underlying the decrease in epithelial cell number, we monitored proliferation and cell survival during pregnancy. There were no apparent differences in apoptosis between Neat1 KO and wild-type glands at any of the time points (pregnancy day 6.5, 8.5, and 12.5) analyzed (data not shown). Immunohistochemistry for Ki67, which stains all but G0 cells, did not reveal differences in the proliferative rate at the start of pregnancy (pregnancy day 6.5; data not shown). In contrast, Neat1^−/−^ alveolar cells exhibited lower rates of proliferation than those observed in Neat1^+/+^ cells at midgestation (8.5 and 12.5 d post-coitum) (Fig. 1H,I). This observation indicates that Neat1-mutant alveolar cells have a decreased ability to sustain high proliferative rates during pregnancy and provides an explanation for the reduced number of luminal alveolar epithelial cells in lactating mammary glands.

### Loss of Neat1 impairs normal mammary gland development and causes ductal and branching morphogenesis defects

**FIGURE 2.**
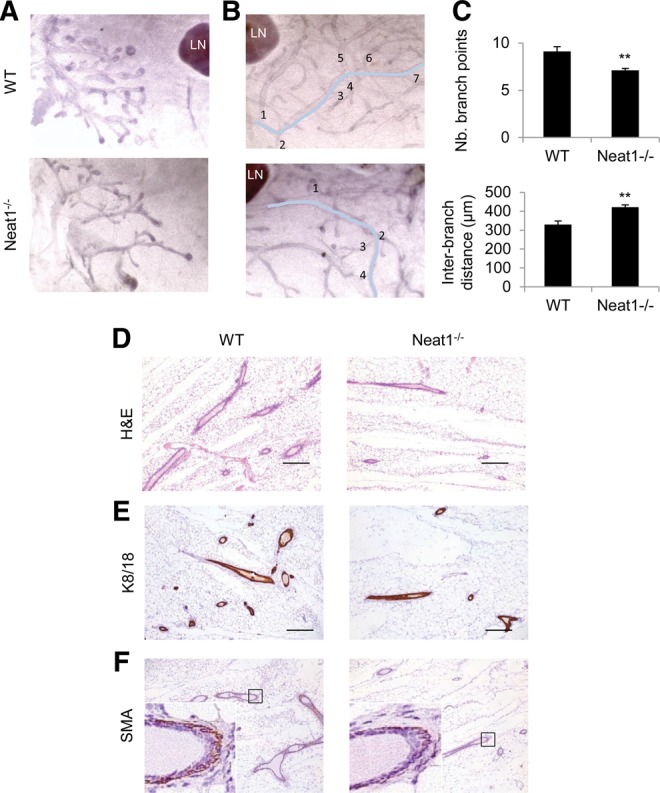
Ductal and branching morphogenesis defects in Neat1 KO females. (*A*) Whole-mount hematoxylin staining of wild-type (WT) and Neat1 KO (Neat1^−/−^) prepubertal inguinal mammary gland. (LN) lymph node. (*B*) Whole-mount hematoxylin staining of wild-type (WT) and Neat1 KO (Neat1^−/−^) adult virgin inguinal mammary gland with indication of counting method for secondary branch points shown in *C*. (LN) Lymph node. (*C*) Average number of secondary branch points over a distance of 3 mm as indicated in *B* and average interbranch distance in micrometer. Statistical significance determined by two-sided *t*-test; (**) *P* = 0.0031 (*upper* panel), (**) *P* = 0.0017 (*lower* panel). (*D*) H&E staining on sections obtained from the inguinal mammary gland contralateral to the ones described in *B*. (*E*) Immunohistochemical staining of cytokeratin 8/18 on consecutive sections of samples in *D*. (*F*) Immunohistochemical staining of α-actin/SMA on consecutive sections of samples in *D*.

We also investigated the importance of Neat1 during post-natal mammary gland development. Development of the mammary gland requires systemic hormones and local growth factors to induce ductal outgrowth and branching (Cowin and Wysolmerski 2010). More specifically, Terminal End Buds (TEBs) form at the tip of ducts, proliferate and ramify into adipose tissue to form the characteristic branched structure of the mammary gland. The glands of Neat1^−/−^ mice show reduced branching at all virgin adult time points analyzed (Fig. 2 and data not shown). At early stages of pubertal development, analysis of whole-mount Neat1^−/−^ mammary outgrowths reveals abnormal branching morphogenesis and aberrant ductal extension compared with littermate Neat1^+/+^ glands (Fig. 2A). By the end of puberty, the primary and secondary ducts of both Neat1^+/+^ and Neat1^−/−^ glands reach the distal edge of the fat pad, but the Neat1^−/−^ glands show decreased density of ducts (Fig. 2B–E). The number of secondary branch points over the same distance of primary duct is significantly decreased, while the interbranch distance is significantly increased in Neat1 KO compared with WT littermates (Fig. 2C). There is clearly less organization and directionality of the branches in the mutant glands as compared with controls. These observations indicate that Neat1 is required for ductal extension and branching morphogenesis in virgin mice.

### Neat1-containing paraspeckles are assembled in luminal epithelial cells of the mammary gland

**FIGURE 3.**
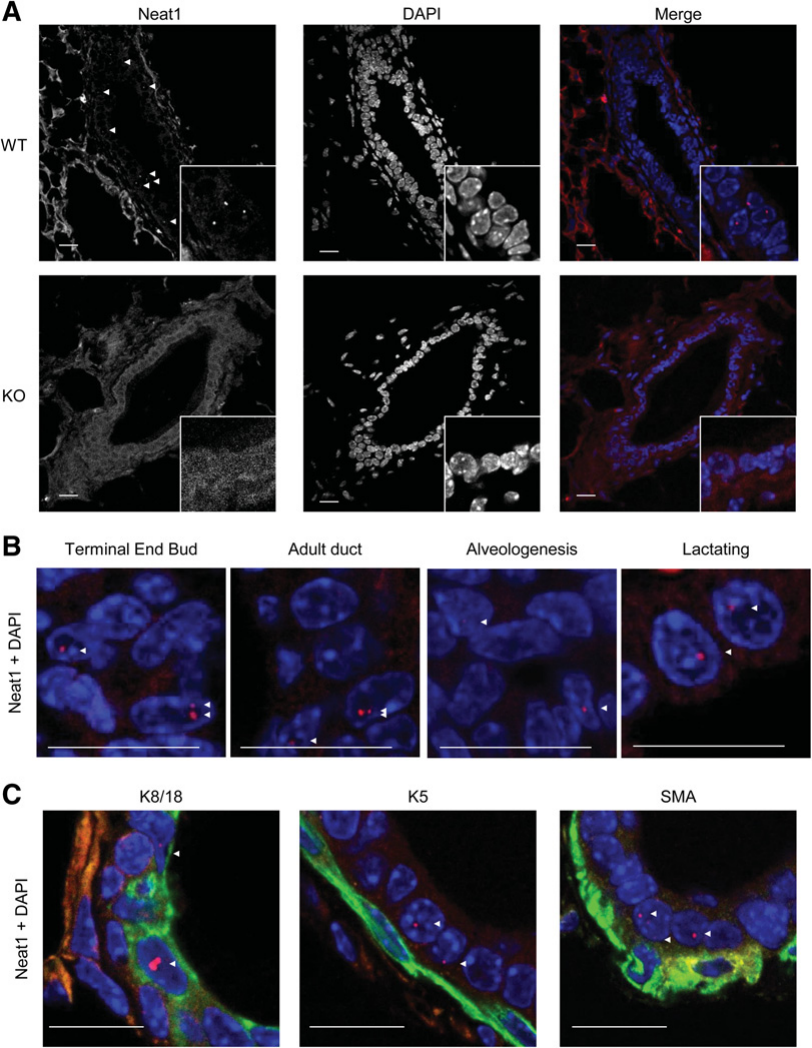
Neat1-paraspeckles are detected in mammary luminal epithelial cells. (*A*) RNA fluorescence in situ hybridization (FISH) of Neat1 (*left* column), with nuclear DAPI counterstain (*middle* column). Wild-type adult virgin duct is represented in the *top* row, Neat1 KO duct *below*. The *right* column shows overlay of DAPI signal (blue) and Neat1 (red). Triangles indicate Neat1-paraspeckles in *left* column with Neat1 signal. (*B*) RNA FISH of Neat1 (red) with DAPI counterstain (blue) throughout post-natal mammary gland development: in terminal end bud of prepubertal 27-d-old animal, in duct of 8- to 9-wk-old adult virgin animal, during alveologenesis at 8.5 d post-coitum, and during lactation at 2 d post-parturition (from *left* to *right*). Triangles indicate Neat1-paraspeckles. (*C*) RNA FISH of Neat1 (red) and immunofluorescent (IF) staining of cell type specific markers (green), with nuclear DAPI counterstain (blue). IF in *left* panel: cytokeratin 8/18, *middle* panel: cytokeratin 5, *right* panel: α-actin/SMA. Triangles indicate Neat1-paraspeckles.

Decreased ductal elongation and branching morphogenesis could be a consequence of loss of Neat1 expression, and possibly paraspeckle formation, in the mammary gland or be the result of secondary dysfunction at distant organ sites. Interestingly, RNA FISH with a probe set specific for Neat1_2 (or with a probe set which detects both Neat1 transcripts) detected the presence of paraspeckles in the mammary gland of virgin WT but not Neat1 KO mice (Fig. 3A). Paraspeckles were also detected in few nuclei of TEB cells and in 30%–50% of K8/18-positive luminal cells in mature ductal structures (Fig. 3B). During alveologenesis, which starts during the first half of pregnancy, although smaller and in reduced numbers, paraspeckles were also detected in 30% of ductal cells. During lactation the Neat1 FISH signal increases and up to 50%–60% of luminal cells show clear Neat1-paraspeckles (Fig. 3B). Paraspeckles were only rarely seen (<0.1% of positive cells) in myoepithelial or stromal cells throughout development (Fig. 3C).

In conclusion, we provide evidence, on the one hand, that Neat1-containing paraspeckles are assembled in luminal epithelial cells of the mammary gland and, on the other hand, that Neat1 is required for both ductal/branching morphogenesis and lobuloalveolar development. As Neat1 is required for paraspeckles formation (Chen and Carmichael 2009; Clemson et al. 2009; Sunwoo et al. 2009), it is tempting to speculate that paraspeckle formation is required in a cell-autonomous fashion for proper mammary gland development and lactation. However, whether Neat1 function is strictly and invariably associated with paraspeckle formation remains to be firmly established. Regardless, this study is the first to assign an important biological function to the lncRNA Neat1 and to link it to the presence of paraspeckles nuclear bodies in vivo.

## MATERIALS AND METHODS

### Mice

Neat1 KO mice and WT littermates are maintained on a pure C57BL/6J background. Female mice were sacrificed at age 27 d (prepubertal stage), at 8–9 wk (adult virgin stage), at 8.5 (and 12.5) d post-coitum during pregnancy, and 2 d after parturition (lactating stage). All animal experiments are approved by ethical committee under project license 089/2013.

### Immunohistochemistry

For histological analysis inguinal mammary fat pads were dissected, stretched on a cardboard support, fixed for 48 h in 10% NBF and processed for paraffin embedding. Samples were then sectioned at 5 µm and routinely stained with hematoxylin (Diapath #C0302) and eosin (Diapath #C0362). Serial sections obtained from the same samples were also immunostained with the following primary antibodies: cytokeratin 8/18 (1/1000, DSHB, TROMA-I, rat monoclonal), α-actin/SMA (1/300, Thermo Scientific #RB-9010, rabbit polyclonal), ki67 (1/200, Thermo Scientific #RM-9106-S, clone SP6, rabbit monoclonal), β-casein (1/1200, Santa Cruz Biotechnology #sc300-42, FL-231, rabbit polyclonal), whey acidic protein (1/4000, Santa Cruz Biotechnology #sc-14832, M-16, goat polyclonal) cleaved-caspase-3 (1/300, Cell Signalling Technology, Asp175, rabbit polyclonal). Slides for immunohistochemistry were deparaffinized in xylene and then rehydrated in ethanol series (100%, 95%, and 70%) and distilled H_2_O. Inhibition of endogenous peroxidase was achieved incubating the slides in 3% H_2_O_2_ for 15 min at RT. Epitope retrieval was performed in citrate buffer (pH6) using 2100 Retriever. Sections were blocked in 1% BSA solution for 40 min at RT and then incubated overnight at +4°C with the primary antibody. For cytokeratin 8/18 and whey acidic protein, biotinylated goat anti-rat secondary antibody (Vector Lab BA-9401) and Vectastain Elite ABC immunoperoxidase kit (PK-6100; Vector Laboratories) were next used according to the manufacturer's protocol. For all the other primary antibodies raised in rabbit, the EnVision+/HRP reagent (Dako K400311) was then applied on sections for 45 min at RT. Immunoreactivity was finally revealed via diaminobenzidine chromogen reaction (Peroxidase substrate kit, DAB, SK-4100; Vector Lab). Next, slides were counterstained in hematoxylin, dehydrated in ethanol series, cleared in xylene, and permanently mounted with a resinous mounting medium (Micromount Diapath, #60200). A 0.1% Tween 20 TBS solution was used as washing buffer in between steps.

For the measurement of cytokeratin 8/18 immunohistochemical expression, three microscopic fields for a total area of 5.63 mm^2^ were randomly selected from the cranial, intermediate, and caudal portion of the lactating mammary fat pad (three animals per genotype). The total immunoreactive area was then calculated in the selected fields applying a digital image analysis algorithm created on the ImageJ software platform. To assess proliferative and apoptotic indexes, Ki67 or cleaved-caspase-3 positive and negative nuclei were counted in three microscopic fields randomly selected from the cranial, intermediate, and caudal portion of the pregnant mammary fat pad at 6.5, 8.5, and 12.5 d post-coitum (three animals per genotype for each of the timepoints were considered). A total average number of 2116 alveolar epithelial cells were analyzed for each mammary sample. Proliferative and apoptotic indexes were then expressed as the ratio between positive and total number of nuclei. The total cell number per image was counted on the ImageJ platform by quantifying DAPI signal in seven randomly selected microscopic fields for a total area of 2.87 mm^2^ per lactating mammary gland.

### RNA fluorescence in situ hybridization on tissue sections

The probes for in situ hybridization (cat: SMF-3010-1) were ordered from Biosearch Technologies, Inc. Cryo and paraffin sections were processed as indicated by the manufacturer. Detailed protocols are available on their website: https://www.biosearchtech.com/stellarisprotocols. For simultaneous immunofluorescence, the following primary antibodies were used: cytokeratin5 (1/1000, Covance, PRB-160P, rabbit polyclonal), cytokeratin8/18 (1/1000, DSHB, TROMA-I, rat monoclonal), and α-actin/SMA (1/300, Thermo Scientific #RB-9010, rabbit polyclonal). Appropriate secondary antibody coupled to Alexa488 (1/500, Life Technologies) are used in washing buffer as detailed in the protocols of Biosearch Technologies, after overnight incubation with RNA probeset and primary antibody.

Imaging was done on a Nikon A1 confocal microscope acquired through a Hercules grant type 1 AKUL/09/037 and images were further processed with ImageJ. For the different developmental stages (prepubertal, virgin adult, 8.5 dpc, 12.5 dpc, and 2 d post-parturition), two to three sections per mammary gland were imaged using groups of three to four animals per genotype.

### Whole-mount hematoxylin staining

The inguinal mammary glands were dissected, mounted onto glass slides and fixed in 4% PFA for at least 24 h, before being transferred to ethanol and xylene for dehydration and clearing. After rehydration, tissues are stained with Mayer's hematoxylin, dehydrated again, and stored in methyl salicylate. Whole mounts were photographed with a LEICA MZ6 microscope and images were transferred to ImageJ for analysis.

Extent of ductal extension was determined based on the average of the length of three straight lines measured from the nipple to the terminal end bud of the three longest ducts invading each mammary gland. Quantification of branch points and the interbranch distance was based on counting the average number of branches originating from three primary ducts (3-mm long each) per mammary gland for three animals of each genotype.
